# Hypothyroidism Affects Uterine Function via the Modulation of Prostaglandin Signaling

**DOI:** 10.3390/ani11092636

**Published:** 2021-09-08

**Authors:** Ilona Kowalczyk-Zieba, Joanna Staszkiewicz-Chodor, Dorota Boruszewska, Krzysztof Lukaszuk, Joanna Jaworska, Izabela Woclawek-Potocka

**Affiliations:** 1 Department of Gamete and Embryo Biology, Institute of Animal Reproduction and Food Research, Polish Academy of Sciences, 10-747 Olsztyn, Poland; j.staszkiewicz-chodor@pan.olsztyn.pl (J.S.-C.); d.boruszewska@pan.olsztyn.pl (D.B.); j.jaworska@pan.olsztyn.pl (J.J.); i.woclawek-potocka@pan.olsztyn.pl (I.W.-P.); 2 Department of Obstetrics and Gynecological Nursing, Faculty of Health Sciences, Medical University of Gdansk, 80-210 Gdansk, Poland; luka@gumed.edu.pl; 3 Department of Obstetrics and Gynecology, The Medical Center of Postgraduate Education, 02-091 Warsaw, Poland; 4 INVICTA Fertility and Reproductive Center, 80-850 Gdansk, Poland

**Keywords:** rat, hypothyroidism, reproduction, prostaglandins, uterine receptivity

## Abstract

**Simple Summary:**

A article proved that, in rats with PTU-induced hypothyroidism, the E2 level as well as the expression of the uterine-receptivity factors homeobox A10 and osteopontin was decreased. Additionally, we observed changes in the expression of PGE_2_, PGF_2α_, and PGI_2_ signaling pathway elements, and changes in the concentrations of those prostaglandins in uterine tissue. The results suggest that hypothyroidism may interfere with the prostaglandin signaling pathway, which may further result in a reduction in uterine receptivity.

**Abstract:**

Thyroid hormones control the functions of almost all body systems. Reproductive dysfunctions, such as abnormal sexual development, infertility, or irregularities in the reproductive cycle, might be associated with thyroid disorders. Uterine receptivity is the period when the uterus is receptive to the implantation of an embryo. During the receptivity period (implantation window), a newly formed blastocyst is incorporated into the uterine epithelium. Prostaglandins are well-known primary mediators of pathological conditions such as inflammation and cancer but are also essential for the physiology of female reproduction. The aim of this study was to evaluate the possible relationship between hypothyroidism and changes in the prostaglandin signaling pathways in the uterus and in the process of uterine receptivity in a rat model. The results show that hypothyroidism impaired uterine receptivity by decreasing the level of E2 as well as decreasing the expression of the uterine-receptivity factors homeobox A10 and osteopontin. Moreover, hypothyroidism caused changes in the expression of elements of the prostaglandin E_2_, F_2α_, and I_2_ signaling pathways and changed the levels of those prostaglandins in the uterine tissue. The results suggest that the mechanisms by which hypothyroidism affects female reproductive abnormalities might involve the prostaglandin signaling pathway, resulting in a subsequent reduction in uterine receptivity.

## 1. Introduction

Thyroid hormones control the functions of almost all the body’s systems. They stimulate growth and development, affect metabolism, and are essential for the proper function of the central nervous system, cardiovascular system, and immune system, as well as influencing the reproductive system [1,2,3,4,5]. Thyroid hormones regulate the secretion of the main reproductive hormones—estradiol (E2) and progesterone (P4). These hormones are necessary for the maturation and development of the oocytes, prepare the endometrium for embryo implantation, and are important in the establishment and maintenance of early pregnancy. It has been shown that, in hypothyroidism, the levels of both E2 and P4 are decreased [5]. Reproductive dysfunction, including abnormal sexual development, infertility, or irregularities in the reproductive cycle, is associated with thyroid disorders [6,7]. It is known that induced hypothyroidism in rats causes a reduction in the absolute volume of the endometrium and a decrease in its muscle layer [7,8]. In humans, hypothyroidism can disrupt the menstrual cycle and ovulation [5,9]. It can also cause problems with fertilization and implantation, miscarriage, and late-pregnancy complications [10,11].

Uterine receptivity is the period when the uterus is receptive to an implanting embryo [12,13]. In rats, the receptivity period occurs between Days 4 and 5 of the estrous cycle [13,14], when the newly formed blastocysts incorporate into the uterine epithelium [13,15]. Estrogen and progesterone are hormones essential for implantation, which is mediated by an increase in the expression of the receptors for those hormones in the endometrium [13,16]. Several molecules are reported to be expressed in the endometrium exclusively during the uterine receptivity period and, therefore, could serve as markers of uterine receptivity [13,17]. Homeobox A10 (HOXA10) is expressed at high levels in adult human and mouse uteruses. Additionally, the significant increase in HOXA10 during the estrous cycle and at the time of implantation [18,19,20] suggests it plays an important role in cyclic endometrial development and uterine receptivity [20]. Other molecules considered as uterine-receptivity markers are osteopontin (OPN) and its receptor, β3 integrin (ITG3B). Both have been found to be coordinately expressed in the human endometrium across the menstrual cycle in fertile women. These glycoproteins are maximally expressed during the implantation window [21,22].

Prostaglandins (PGs) are biologically active lipids. They are well-known primary mediators of pathological conditions such as inflammation and cancer, but are also essential for the physiology of female reproduction [23]. PGs belong to the group of prostanoids that are generated from arachidonic acid (AA). This acid is converted to PGH_2_ with the participation of prostaglandin endoperoxide synthases (PTGSs). There are two main isoforms of prostaglandin endoperoxide synthases, PTGS1 and PTGS2 [24,25]. PGE_2_, PGF_2α_, and PGI_2_ are synthesized from PGH_2_ by PGE synthases (PTGES-1, PTGES-2, and PTGES-3), PGF synthase (PGFS), and PGI synthase (PGIS) [25,26]. Prostaglandins act by interacting with specific G-protein-coupled receptors [27,28,29]. PGE_2_ transduces signals through four types of receptors—PTGER1, 2, 3, and 4 (EP1, 2, 3, and 4)—while PGF_2α_ acts through PTGFR (FP) [29,30,31]. PGI_2_ acts through PTGIR (IP) [25,32], but it can also act via the peroxisome-proliferator-activated receptors PPARα, PPARγ, and PPARδ, which are members of the nuclear-hormone-receptor superfamily [25,33].

PGE_2_ and PGF_2α_ are very important factors in female reproduction. They are involved in blastocyst spacing, implantation, and decidualization, as well as in uterine contraction [29,31,34]. EP1, EP3, and FP affect smooth muscle contraction, while EP2 and EP4 affect the relaxation of smooth muscles [29,32,35]. The expression of EPs and FP in the human uterus varies during the menstrual cycle. EP1 dominates in the early-secretory phase, while EP2, EP3, and EP4 dominate in the mid-secretory phase, and FP, in the proliferative phase [29,36]. In pigs, the inhibition of the synthesis of PGs by blocking the activity of PTGS2 causes pregnancy loss [25,37]. An appropriate ratio between the luteoprotective PGE_2_ and the luteolytic PGF_2α_ is very important for the successful establishment of pregnancy in pigs [25,38]. PGI_2_ is the most abundant prostanoid produced by the endometrium of mice and cattle. In mice, prostacyclin is critical for endometrial decidualization and embryo implantation. In rodents, cattle, and sheep, it has been demonstrated that signaling involving PGI_2_ and its receptor is an important component of the embryo–uterus interactions that are essential for successful implantation. PGI_2_ and PTGIR signaling are very important components of embryo–uterus interactions that are essential for successful implantation [25,39,40,41,42,43]. Furthermore, PGI_2_ increases embryonic cell proliferation and reduces apoptosis [25,44,45]. It also enhances the embryo hatching and live birth potential of mouse embryos [25,46,47].

The role of prostaglandins in embryo implantation is indisputably essential [48,49]. Poor endometrial receptivity during embryo implantation has been linked to reduced prostaglandin synthesis in the human endometrium [50]. On the other hand, it is also known that hypothyroidism can cause problems with fertilization and implantation, miscarriages, and late-pregnancy complications [10,11]. The aim of this study was to evaluate the possible relationship between PTU-induced hypothyroidism and changes in prostaglandin signaling pathways in the uterus and in the process of uterine receptivity in a rat model.

## 2. Materials and Methods

### 2.1. Animals

All the experimental procedures were approved by the Local Animal Care and Use Committee in Olsztyn, Poland (Agreement No. 40/2015/DTN).

Female Wistar rats aged 8–10 weeks were kept in the Animal Laboratory of the Institute of Animal Reproduction and Food Research of the Polish Academy of Sciences in Olsztyn. The rats were conventionally housed. They were divided into two groups: the control group (*n* = 20) and the experimental group with induced hypothyroidism (*n* = 20). The control group was fed ad libitum. Over the 90 days, the study group, besides the normal diet, also received 0.05% 6-propyl-2-thiouracil solution (PTU) (#46698-250MG, Sigma-Aldrich, Munich, Germany) by oral administration to induce hypothyroidism. The duration of the PTU treatment was selected based on a study by Jena and Bhanja [51]. Then, the animals were sacrificed. The blood serum and uterus were collected and immediately frozen. The tissues were stored at −80 °C until mRNA and protein extraction.

### 2.2. Confirmation of Hypothyroidism

To confirm hypothyroidism, the thyroid hormone index (T4, T3, and thyrotropin (TSH)) for the blood serum samples was obtained. Serum T3 (#EKU04275), T4 (#EKU04274), and TSH (#EKC39776) measurements were performed using ELISA kits according to the manufacturer’s instructions (Biomatik, ON, Canada).

### 2.3. mRNA Isolation and Real-Time PCR

mRNA was isolated using the Total RNA Mini Plus Kit (#036-100; A&A Biotechnology; Gdansk, Poland). The quality and quantity of the mRNA were measured using the NanoDrop 100 (Thermo Fisher Scientific; Waltham, MA, USA). Reverse transcription was performed using the Maxima First Strand cDNA Synthesis Kit for RT-qPCR (#K1642; Thermo Fisher Scientific; Waltham, MA, USA). Real-time PCR was performed with the ABI Prism 7900 (Applied Biosystems, Life Technologies, Waltham, MA, USA) sequence detection system using the Maxima SYBR Green/ROX qPCR Master Mix (#K0223; Thermo Fisher Scientific, Waltham, MA, USA). PCRs were performed in 384-well plates. The results for the mRNA transcription were normalized to β-actin (ACTB, internal control). The mRNA levels are shown in arbitrary units. The primers were designed using Primer3web version 4.0.0 (http://primer3.ut.ee; accessed on 23 April 2019), and their sequences are shown in Table 1. For the relative quantification of the mRNA levels, the Miner software was used (http://www.miner.ewindup.info; accessed on 23 April 2019).

### 2.4. Protein Isolation and Western Blotting

Total protein (*n* = 7) was isolated using the Radio Immuno Precipitation Assay Buffer (150 mM NaCl, 50 mM Tris, 0.1% SDS, 1% Triton × 100, 0.5% sodium deoxycholate, and 5 mM EDTA; pH = 7.2). The protein concentration was measured using the Micro BCA method. The levels of the proteins involved in prostaglandin signaling pathways—PTGS2, PTGES-2, PTGES-3, PTGER1, PTGER2, PTGER3, PTGER4, PGFS, PTGFR, PTGIS, and PTGIR—and the uterine-receptivity proteins HOXA10 and OPN were measured by Western blotting using the semi-dry method of transfer (Trans-Blot SD Cell, Bio-Rad; California, USA) onto polyvinylidene difluoride membranes (Immobilon-P Transfer Membrane; #IPVH00010, Millipore; Burlington, MA, USA). The primary antibodies used are shown in Table 2. β-actin antibody (#A2228-100UL, Sigma-Aldrich; Munich, Germany), diluted 1:4000, was used as the internal control for protein loading. The secondary antibodies used were goat anti-mouse IgG (whole molecule)–alkaline phosphatase (#A3562, Sigma-Aldrich; Munich, Germany), diluted 1:30,000, and goat anti-rabbit IgG–AP (#sc-2007, Santa Cruz, TX, USA), diluted 1:5000. The immune complexes were visualized using the alkaline phosphatase visualization procedure. The blots were scanned for densitometric analyses (Versa Doc Imagine System), and the specific bands were quantified using the Image Lab Software Version 5.2 (Bio-Rad Laboratories, CA, USA).

### 2.5. The Extraction of Prostaglandins and the Measurement of Their Concentrations in the Uterine Tissue

PGE_2_, PGF_2α_, and PGI_2_ were extracted from the uterine tissue using diethyl ether (#384210114, POCH, Gliwice, Poland). The concentrations of PGE_2_, PGF_2α_, and PGI_2_ were measured using the PGE2 high-sensitivity ELISA kit (#ADI-931-069; ENZO Life Sciences Inc., New York, NY, USA), the PGF2α high-sensitivity ELISA kit ((#ADI-931-069; ENZO Life Sciences Inc., New York, NY, USA), and the urinary prostacyclin ELISA kit (#ADI-901-025, ENZO Life Sciences Inc., New York, NY, USA), respectively.

### 2.6. Statistical Analyses

Statistical analyses were conducted using GraphPad Prism 7 (GraphPad Software, Inc., CA, USA). All the data are shown as the mean ± SEM, and differences were considered to be significantly different at a 95% confidence level (*p* < 0.05). Analyses were performed using Student’s *t*-tests.

## 3. Results

### 3.1. The Levels of Thyroid Hormones in the Blood Serum Samples

**Figure 1:**
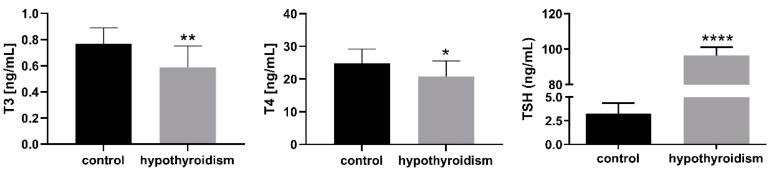
The levels of T3, T4, and TSH in blood serum samples (ng/mL). The data are presented as the mean ± SEM. Asterisks indicate significant differences ((* *p* ≤ 0.05, ** *p* ≤ 0.01, *** *p* ≤ 0.001, **** *p* ≤ 0.0001), as determined by Student’s *t*-test.

Figure 1 shows the T3, T4, and TSH levels in the blood serum samples in the control group and the experimental group with PTU-induced hypothyroidism. The levels of T3 and T4 were significantly decreased (*p* < 0.05), whereas the level of TSH was significantly increased (*p* < 0.05) in the blood serum samples from the experimental group in comparison to the control group.

### 3.2. Uterine Receptivity

**Figure 2:**
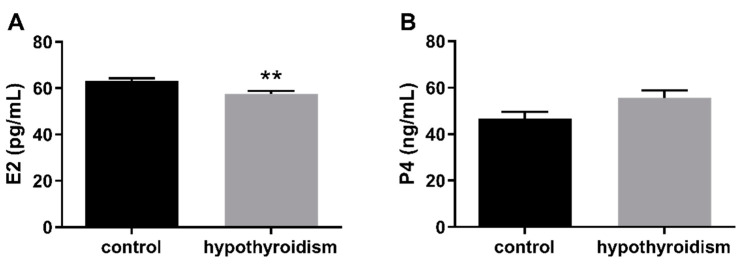
The levels of (**A**) E2 (pg/mL) and (**B**) P4 (ng/mL) in blood serum samples. The data are presented as the mean ± SEM. Asterisks indicate significant differences (* *p* ≤ 0.05, ** *p* ≤ 0.01, *** *p* ≤ 0.001, **** *p* ≤ 0.0001), as determined by Student’s *t*-test.

Figure 2 shows the E2 and P4 levels in the blood serum samples in the control group and the experimental group with PTU-induced hypothyroidism. The level of E2 was significantly decreased (*p* < 0.05), whereas that of P4 was not changed (*p* > 0.05) in the blood serum samples from the experimental group in comparison to the control group.

**Figure 3:**
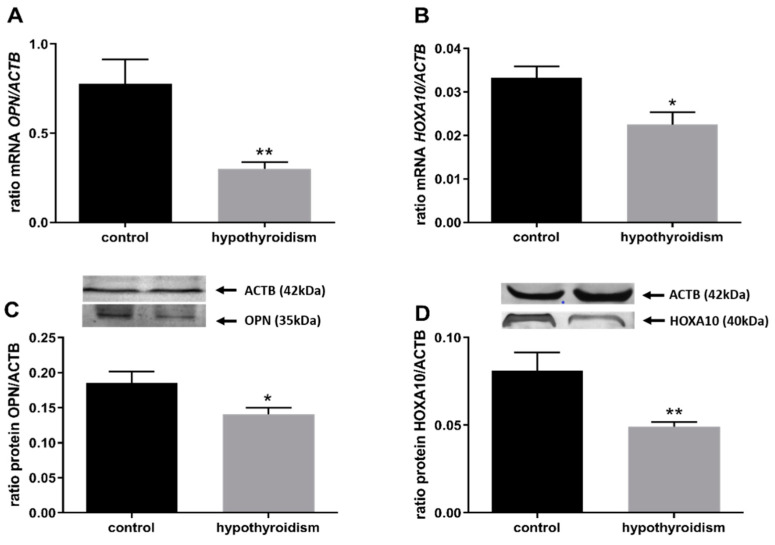
The effects of hypothyroidism on the mRNA and protein abundance of uterine-receptivity factors in the uterine tissue of rats: (**A**,**C**): OPN/OPN; (**B**,**D**): HOXA10/HOXA10. The values are presented as arbitrary units and expressed as the mean ± SEM. Stars indicate significant differences (* *p* ≤ 0.05, ** *p* ≤ 0.01, *** *p* ≤ 0.001, **** *p* ≤ 0.0001), as determined by Student’s *t*-test.

Figure 3 shows mRNA and protein expression patterns of the uterine-receptivity factors osteopontin (OPN/OPN) and homeobox A10 (HOXA10/HOXA10). In both cases, the mRNA and protein expression levels were significantly lower in the group of rats with hypothyroidism than the control group (*p* < 0.05).

### 3.3. Prostaglandin Signaling in the Uterine Tissue

**Figure 4:**
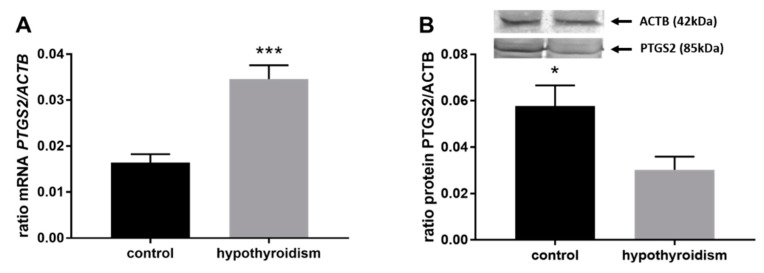
The effect of hypothyroidism on the mRNA (**A**) and protein (**B**) abundance of prostaglandin-endoperoxide synthase 2 in the uterine tissue of rats. The values are presented in arbitrary units and expressed as the mean ± SEM. Stars indicate significant differences (* *p* ≤ 0.05, ** *p* ≤ 0.01, *** *p* ≤ 0.001, **** *p* ≤ 0.0001), as determined by Student’s *t*-test.

**Figure 5:**
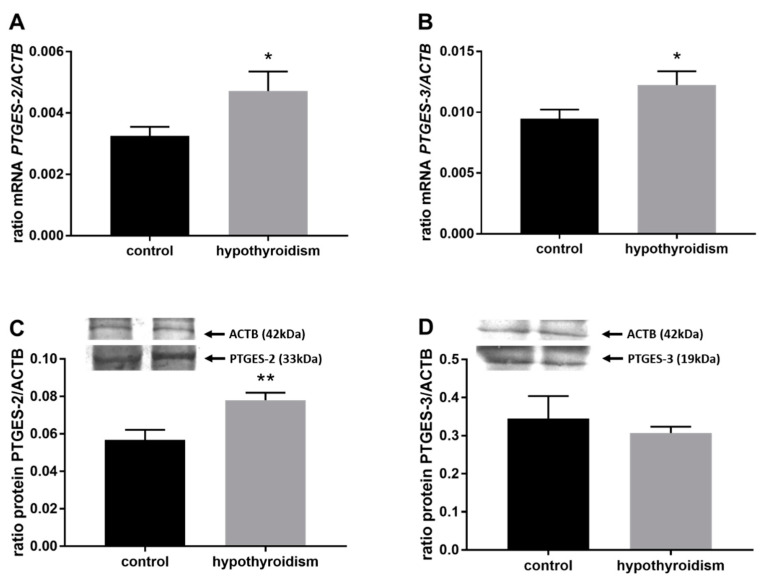
The effects of hypothyroidism on the mRNA and protein abundance of PGE_2_ synthase 2 in the uterine tissue of rats: (**A**,**C**): PTGES-2/PTGES-2; (**B**,**D**): PTGES-3/PTGES-3. The values are presented in arbitrary units and expressed as the mean ± SEM. Stars indicate significant differences (* *p* ≤ 0.05, ** *p* ≤ 0.01, *** *p* ≤ 0.001, **** *p* ≤ 0.0001), as determined by Student’s *t*-test.

**Figure 6:**
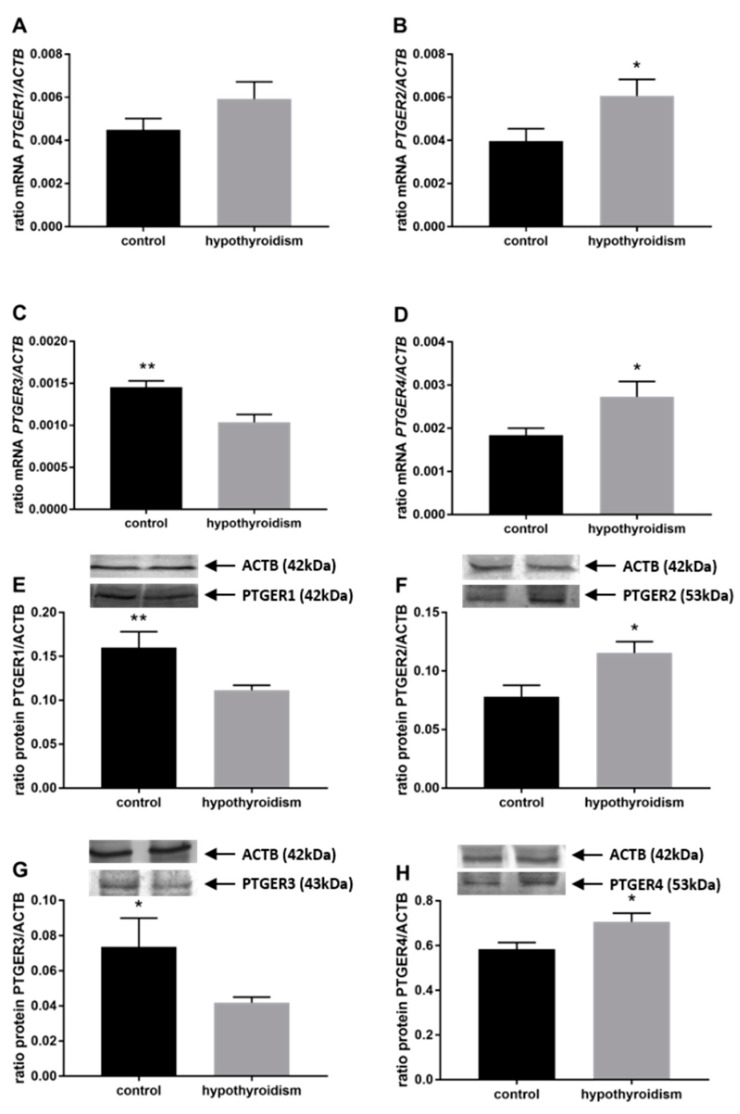
The effects of hypothyroidism on the mRNA and protein abundance of PGE_2_ receptors in the uterine tissue of rats: (**A**,**E**): PTGER1/PTGER1; (**B**,**F**): PTGER2/PTGER2; (**C**,**G**): PTGER3/PTGER3; (**D**,**H**): PTGER4/PTGER. The values are presented in arbitrary units and expressed as the mean ± SEM. Stars indicate significant differences (* *p* ≤ 0.05, ** *p* ≤ 0.01, *** *p* ≤ 0.001, **** *p* ≤ 0.0001), as determined by Student’s *t*-test.

**Figure 7:**
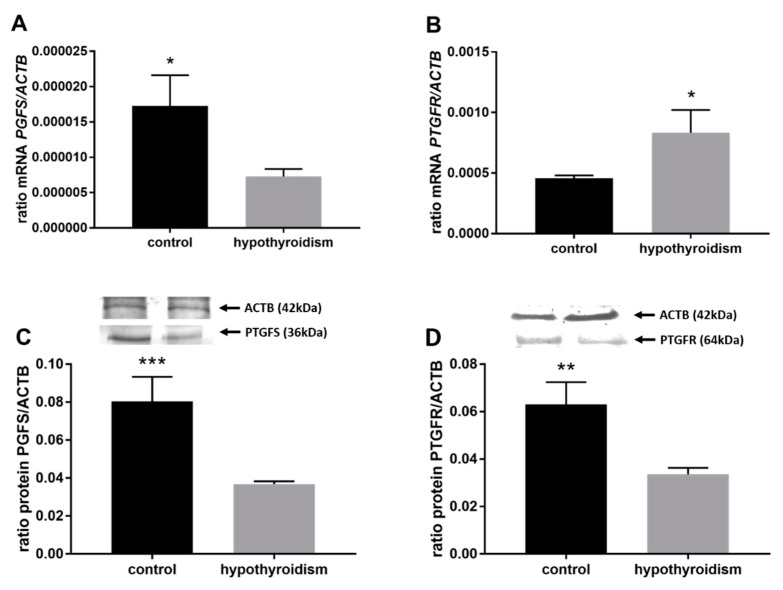
The effects of hypothyroidism on the mRNA and protein abundance of PGF_2α_ synthase and its receptor in the uterine tissue of rats: (**A**,**C**): PGFS/PGFS; (**B**,**D**): PTGFR/PTGFR. The values are presented in arbitrary units and expressed as the mean ± SEM. Stars indicate significant differences (* *p* ≤ 0.05, ** *p* ≤ 0.01, *** *p* ≤ 0.001, **** *p* ≤ 0.0001), as determined by Student’s *t*-test.

**Figure 8:**
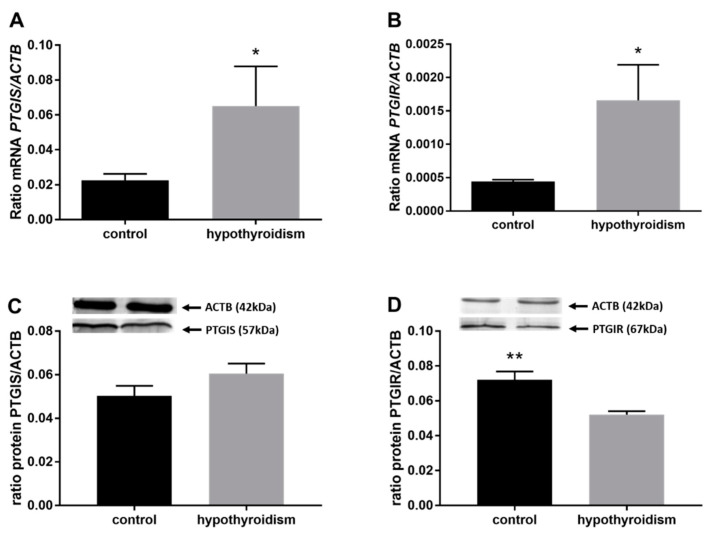
The effects of hypothyroidism on the mRNA and protein abundance of PGI_2_ synthase and its receptor in the uterine tissue of rats: (**A**,**C**): PGIS/PGIS; (**B**,**D**): PTGIR/PTGIR. The values are presented as arbitrary units and expressed as the mean ± SEM. Stars indicate significant differences (* *p* ≤ 0.05, ** *p* ≤ 0.01, *** *p* ≤ 0.001, **** *p* ≤ 0.0001), as determined by Student’s *t*-test.

Figure 4 shows the mRNA and protein expression patterns of prostaglandin endoperoxide synthase 2 PTGS2/PTGS2 (COX-2). The mRNA level of PTGS2 was significantly higher in the group with hypothyroidism than the control group, while the protein level of PTGS2 was significantly lower in the group with hypothyroidism. Figure 5 shows the mRNA and protein expression patterns of PGE_2_ synthases. The mRNA and protein levels of PTGES-2/PTGES-2 were significantly higher in the experimental group with induced hypothyroidism than the control group (*p* < 0.05). There was significantly higher PTGES-3 mRNA expression in the group of animals with induced hypothyroidism (*p* < 0.05), while its protein expression was maintained at the same level in both groups. Figure 6 shows the mRNA and protein expression patterns of the PGE_2_ receptors. There was no significant difference in the mRNA expression of PTGER1 between the groups, whereas the expression of the PTGER1 protein was significantly lower in the experimental group with induced hypothyroidism than the control group (*p* < 0.05). The mRNA and protein expression levels of PTGER2/PTGER2 and PTGER3/PTGER3 show similar patterns. The expression levels were significantly lower in the group of animals with hypothyroidism than the control group of rats (*p* < 0.05). The mRNA and protein expression levels of PTGER4/PTGER4 were significantly higher in the experimental group in comparison to the control group of rats (*p* < 0.05). Figure 7 shows the mRNA and protein expression patterns of PGF_2α_ synthase PGFS/PGFS and the receptor for PGF_2α_ (PTGFR/PTGFR). The PGFS/PGFS expression pattern was similar for both mRNA and protein. It was significantly lower in the group of animals with hypothyroidism than the control group (*p* < 0.05). The mRNA and protein expression patterns of the PGF_2α_ receptor PGFR/PGFR were opposite. The mRNA level was significantly higher, while the protein level was significantly lower, in the group of animals with hypothyroidism than the control group (*p* < 0.05). Figure 8 shows the mRNA and protein expression patterns of prostacyclin synthase PTGIS/PTGIS and the prostacyclin receptor PTGIR/PTGIR. The mRNA level of PTGIS was significantly higher in the group of rats with induced hypothyroidism than the control group of animals, while there was no difference in the protein level of PTGIS between the groups. In the case of the prostacyclin receptor, the mRNA level of PTGIR was significantly higher in the experimental group of animals than the control group, whereas the protein level of PTGIR was significantly lower in the group of rats with hypothyroidism than the control group (*p* < 0.05).

### 3.4. The Concentrations of Prostaglandins in the Uterine Tissue

**Figure 9:**
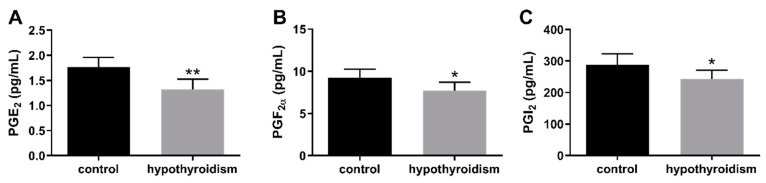
The effect of hypothyroidism on the concentrations (pg/mL) of PGs in uterine tissue of rats: (**A**): PGE_2_; (**B**): PGF_2α_; (**C**): PGI_2_. The values are presented in arbitrary units and expressed as the mean ± SEM. Stars indicate significant differences (* *p* ≤ 0.05, ** *p* ≤ 0.01, *** *p* ≤ 0.001, **** *p* ≤ 0.0001), as determined by Student’s *t*-test.

Figure 9 shows the concentrations of PGs in the uterine tissue. The concentrations of PGE_2_, PGF_2α_, and PGI_2_ in the uterine tissue were significantly lower in the experimental group of animals with induced hypothyroidism than the control group of animals (*p* < 0.05).

## 4. Discussion

Our present study provides a new understanding of the possible relationship between thyroid hormones and uterine receptivity, as well as prostaglandin signaling pathways in the rat uterus. To the best of our knowledge, this is the first report to investigate the effect of PTU-induced hypothyroidism on uterine receptivity and the uterine synthesis of prostaglandins in rats.

Hypothyroidism is a disease characterized by a defect in the production of thyroid hormones due to the insufficient stimulation by TSH of normal thyroid gland function. This condition is the consequence of an anatomic or functional disorder of the pituitary gland or the hypothalamus, resulting in variable alterations of TSH secretion [52]. Other causes include a congenital absence of the thyroid, radioiodine therapy for hyperthyroidism, surgical thyroidectomy, various drugs that affect thyroid function or cause thyroid inflammation, and a variety of other rarer causes [53]. Congenital hypothyroidism occurs in ~1/4000 infants [54]. The lifetime risk of overt hypothyroidism is around 5%, and this disease is usually preceded by subclinical hypothyroidism, which has an even higher prevalence (estimated to be up to 9%) [52].

It is known from the literature that thyroid hormones affect reproductive capacity and, in particular, play an important role during implantation and the early stages of embryo development. Successful implantation is the result of reciprocal interactions between the implantation-competent blastocyst and the receptive uterus [55]. Thus, thyroid hormones may also influence angiogenesis and immune function in the uterus during implantation. Angiogenesis is crucial for successful implantation, decidualization, and placentation. In the case of immune function, natural killer (NK) cells have emerged as crucial modulators of implantation and placental angiogenesis. It is known that NK cell concentrations are higher in patients with thyroid autoimmunity and recurrent spontaneous abortion or unexplained infertility. This suggests their involvement in the decrease in uterine receptivity and the increase in pregnancy loss. Thyroid hormones have been reported to significantly influence the concentrations of the female reproductive hormones estradiol (E2) and progesterone (P4) [5,11,56]. In the current study, we observed that the administration of PTU in the drinking water decreased the circulating E2 level compared to control. We did not observe any effect on the P4 level. Our results are partially consistent with the results of Kong et al. [5] and Tohei [57], who found that, in rats with hypothyroidism, the levels of both E2 and P4 were decreased. However, Hapon et al. [58] found significantly higher luteal P4 content on Day 21 of pregnancy in rats with hypothyroidism compared to control, suggesting that hypothyroidism impairs CL function during gestation, delaying the onset of parturition in the rat. As P4 and E2 are necessary for the maturation and development of oocytes, preparing the endometrium for embryo implantation, and are important in the establishment and maintenance of early pregnancy, changes in the physiological levels of these hormones caused by TH hormones may contribute to disorders of or disrupt the reproductive system. Furthermore, in our experiment, in the groups of animals with PTU-induced hypothyroidism, the levels of homeobox A10 (*HOXA10*/HOXA10) and osteopontin (*OPN*/OPN), which are considered to be uterine receptivity markers [13], were significantly lower in comparison to those in the control group. Hoxa10/HOXA10 has been found to be directly involved in embryogenesis and embryo implantation via the regulation of downstream genes. The cyclical endometrial expression of this factor, with a peak of expression occurring during the window of implantation, is observed in response to E2 and P4 [59]. Osteopontin, a member of the extracellular matrix (ECM) protein family, is involved in many physiological and pathological processes, including cell adhesion, cell proliferation and differentiation, angiogenesis, and tumor metastasis. Weintraub et al. [60] showed that OPN-deficient mice manifested a decreased pregnancy rate during mid-gestation and that the knockdown of OPN in mouse endometrial stromal cells restrained trophoblast invasion in vitro. Additionally, OPN was proved to be activated in mouse endometrial stromal cells (mESCs) and human endometrial stromal cells (hESCs) by E2 and P4 [61,62]. These results suggest that OPN plays an important role in regulating blastocyst implantation and decidualization. Our data confirm that, in hypothyroidism, uterine receptivity is reduced. A disturbed E2 level and decreased expression of HOXA10 and OPN may result in difficulties with the implantation of blastocysts and the maintenance of pregnancy.

Prostaglandin endoperoxide synthase 2 (PTGS2) is involved in inflammation and in essential reproductive processes, including ovulation, fertilization, implantation, and decidualization. PTGS2-deficient females are infertile, with abnormalities in ovulation, fertilization, implantation, and decidualization [63,64]. PTGS2 is the key enzyme involved in prostaglandin synthesis [65,66]. Gillio-Meina et al. [42] showed that the PTGS2 protein is gradually expressed in the rat endometrium in response to deciduogenic stimuli. In our study, we showed that the PTGS2 protein level was significantly lower in the group of rats with induced hypothyroidism in comparison to the control group of animals. Combining that information with the knowledge that induced hypothyroidism in rats caused a reduction in the absolute volume of the endometrium [7,8], it may be speculated that the decreased level of PTGS2 in the uterus in the group of rats with induced hypothyroidism was caused by the significantly reduced volume of endometrium.

PTGES-2 and PTGES-3 are responsible for the conversion of PGH_2_ to PGE_2_, while PGFS converts PGH_2_ to PGF_2α_. PGIS is responsible for the conversion of PGH_2_ to PGI_2_. These enzymes are involved in the reproductive processes [25,26]. Their expression at implantation sites in rats and other species has been reported [48,49]. In our study, we showed higher expression levels of PTGES-2 in the experimental group of animals, whereas the expression of PTGES-3 and PGIS was at the same level in the experimental and control groups. The expression of PGFS was significantly lower in the group of rats with hypothyroidism. This suggests that hypothyroidism may stimulate the synthesis of PTGES2 and inhibit the synthesis of PGFS but does not affect the synthesis of PTGES-3 or PGIS. In the present study, the mRNA and protein expression patterns of PGFR as well as PGIR were opposite. The mRNA level was significantly higher, while the protein level was significantly lower in the group of animals with hypothyroidism in comparison to the control group. This could be a result of post-translational changes in proteins. These changes regulate the functional activities of proteins in cells and their interactions with other cellular molecules. It is considered that this modification plays a key role in modulating protein function.

It has been reported that the prostaglandin receptors EP1, EP3, and FP promote uterine smooth muscle contraction, while EP2 and EP4 affect uterine smooth muscle relaxation [29,34,35]. Blesson et al. [29] also indicated that the expression levels of the receptors of PGs are regulated by steroid hormones. In the human endometrium, it has been found that the levels of PG receptors vary in a phase-specific manner. EP2, EP3, and EP4 dominate in the mid-secretory phase, while EP1 dominates in the early-secretory phase, and FP dominates in the proliferative phase. This suggests that the expression of those receptors may be regulated by E2 and P4 [29,36]. Our study showed that induced hypothyroidism caused lower expression levels of EP1, EP3, and FP and higher expression levels of EP2 and EP4. It suggests that hypothyroidism leads to the impairment of the uterus’ function and disturbances of the estrous cycle by inducing changes in the expression levels of PG receptors in the uterine tissue. Furthermore, EP2, EP3, and EP4 might participate in the regulation of stromal edema, endometrial blood flow, and blood vessel permeability [29,36]. This, in turn, indicates that hypothyroidism can cause abnormalities in the cyclic changes in the uterine blood supply via changes in the expression of those receptors in the rat uterus. It is known that, in rodents, PGI_2′_s signaling and the expression of its receptor (PTGIR) are very important components of embryo–uterus interactions and are essential for successful implantation [25,39,41,42]. In mice, PGI_2_ is also critical for endometrial decidualization and embryo implantation [25,39]. In addition, PGI_2_ increases embryonic cell proliferation and reduces apoptosis [25,44,45]. It also enhances embryo hatching and the live birth potential of mouse embryos [25,46,47]. In our study, we also demonstrated a lower level of PTGIR in the group of animals with induced hypothyroidism. This implies that hypothyroidism may cause changes in endometrial decidualization, leading to difficulties in embryo implantation and the maintenance of pregnancy.

## 5. Conclusions

In summary, this study is the first to characterize the influence of hypothyroidism on uterine receptivity and PG signaling in uterine tissue in a rat model. The results show that hypothyroidism impairs uterine receptivity by decreasing the level of E2 as well as the expression of the uterine-receptivity factors HOXA10 and OPN. Hypothyroidism also causes changes in the expression of elements of the PGE_2_, PGF_2α_, and PGI_2_ signaling pathways, and changes in the concentrations of those prostaglandins in uterine tissue. In general, it decreases the expression of those factors in the rat uterus. It is known that prostaglandins are strongly involved in uterine functions, such as decidualization and blastocyst implantation, and therefore, hypothyroidism causes abnormalities in the female reproductive system. Our results suggest that hypothyroidism may interfere with the prostaglandin signaling pathway, which may further result in a reduction in uterine receptivity. However, this hypothesis requires further investigation.

## Figures and Tables

**Table 1:** Primers used for real-time PCR.

Gene Symbol	GeneBank Accession No.	Forward Primer Reverse Primer	Primer Size (bp)
PTGS2	NM_017232	5′-AAAGGCCTCCATTGACCAGA-3′ 5′-TCGATGTCATGGTAGAGGGC-3′	20 20
PTGES2	NM_001107832.1	5′-AAAGGAAGCCAGGACGGAGGA-3′ 5′-CCTCGGCAGGTGTTCGGT-3′	21 18
PTGES3	M_001130989.1	5′-GCTGCCGGAGAGGAGTCG-3′ 5′-AGGCTGCATGGTGAACGGG-3′	18 19
PTGER1	M_001278475.1	5′-GCTCCCTGCCTTTCACAATCT-3′ 5′-TCTCAGGACTGGTGGTCTAAGGA-3′	21 23
PTGER2	NM_031088.1	5′-GAAAGGACTTCTATGGCGGAGG-3′ 5′-AAGCAAAGATTGTGAAAGGCAGG-3′	22 24
PTGER3	NM_012704.1	5′-CGCAGATGGGAAAGGAGAAGGA-3′ 5′-AGGTTGTTCATCATCTGGCAGAACT-3′	22 25
PTGER4	NM_032076.3	5′-CATTCCCGCTCGTGGTGCGA-3′ 5′TCTGCTGATGGTCTTTCACCACAC-3′	19 23
PGFS	NM_138510.1	5′-GGTATCTCTGAAGCCAGGGGA-3′ 5′-TTGGACACCCCGATGGACTTG-3′	21 21
PTGFR	NM_013115.1	5′-CCCTTTCTGGTGACGATGGC-3′ 5′-TCCGTAGCAGAATGTAGACCCA-3′	20 22
PTGIS	NM_031557.2	5′-GGGCCTCCTGACTTCCTGTTG-3′ 5′-AGCTTTTCCTGCTCTCGGTGT-3′	21 21
PTGIR	NM_001077644.1	5′-TCCCTGCCTCTCACGATCAG-3′ 5′-AAAACGGAAGGCGTGGAGGT-3′	20 20
OPN	AB001382.1	5′-TGAAAGTGGCTGAGTTTGGC-3′ 5′-TCGTCGTCATCATCGTCCAT-3′	20 20
HOXA10	NM_001129878.1	5′-CATTCAGGCCCCATCTCAGA-3′ 5′-TTCAGCCCCTCATAGCCAAA-3′	20 20
ACTB	KJ696744.1	5′-CCACACCCGCCACCAGTTCG-3′ 5′-CTAGGGCGGCCCACGATGGA-3′	20 20

**Table 2:** Antibodies used for Western blotting.

Protein	Catalog N°	Host	Dilution	Predicted Molecular Weight (kDa)
PTGS-2	Santa Cruz, sc-7951	Rabbit	1:100	85
PTGES-2	Cayman, 160145	Rabbit	1:200	33
PTGES-3	Abcam, ab92503	Rabbit	1:10,000	19
PTGER1	Abcam, ab183073	Rabbit	1:3000	42
PTGER2	Abcam, ab16151	Mouse	1:200	53
PTGER3	Abcam, ab16152	Mouse	1:100	43
PTGER4	Santa Cruz, sc-20677	Rabbit	1:100	53
PGFS	Abcam, ab84327	Rabbit	1:500	36
PTGFR	Cayman, 101802	Rabbit	1:200	64
PTGIS	Abcam, ab23668	Rabbit	1:250	57
PTGIR	Cayman, 10005518	Rabbit	1:200	67
OPN	Abcam ab8448	Rabbit	1:1000	66
HOXA10	Abcam ab23392	Rabbit	1:1000	42
ACTB	SigmaAldrich A2228	Mouse	1:4000	42

## Data Availability

The data that support the findings of this study are available on request from the corresponding author, I.K.-Z.
